# GMM-HMM-Based Eye Movement Classification for Efficient and Intuitive Dynamic Human–Computer Interaction Systems

**DOI:** 10.3390/jemr18040028

**Published:** 2025-07-09

**Authors:** Jiacheng Xie, Rongfeng Chen, Ziming Liu, Jiahao Zhou, Juan Hou, Zengxiang Zhou

**Affiliations:** 1 Department of Precision Machinery and Precision Instrumentation, University of Science and Technology of China, Hefei 230027, China; jc_xie@mail.ustc.edu.cn (J.X.); crf0114@mail.ustc.edu.cn (R.C.); lzm1224@mail.ustc.edu.cn (Z.L.); jh152728@mail.ustc.edu.cn (J.Z.); 2 Department of Psychology, School of Philosophy, Anhui University, Hefei 230039, China; daisyhoujuan@gmail.com

**Keywords:** eye movements, human–computer interaction, assistive robotic arm, GMM-HMM

## Abstract

Human–computer interaction (HCI) plays a crucial role across various fields, with eye-tracking technology emerging as a key enabler for intuitive and dynamic control in assistive systems like Assistive Robotic Arms (ARAs). By precisely tracking eye movements, this technology allows for more natural user interaction. However, current systems primarily rely on the single gaze-dependent interaction method, which leads to the “Midas Touch” problem. This highlights the need for real-time eye movement classification in dynamic interactions to ensure accurate and efficient control. This paper proposes a novel Gaussian Mixture Model–Hidden Markov Model (GMM-HMM) classification algorithm aimed at overcoming the limitations of traditional methods in dynamic human–robot interactions. By incorporating sum of squared error (SSE)-based feature extraction and hierarchical training, the proposed algorithm achieves a classification accuracy of 94.39%, significantly outperforming existing approaches. Furthermore, it is integrated with a robotic arm system, enabling gaze trajectory-based dynamic path planning, which reduces the average path planning time to 2.97 milliseconds. The experimental results demonstrate the effectiveness of this approach, offering an efficient and intuitive solution for human–robot interaction in dynamic environments. This work provides a robust framework for future assistive robotic systems, improving interaction intuitiveness and efficiency in complex real-world scenarios.

## 1. Introduction

Human–computer interaction (HCI) has become a central focus in the development of advanced technologies, aiming to create seamless communication between humans and machines [1]. Among various input modalities, eye-tracking technology stands out as an intuitive and non-invasive method that enables direct and natural interaction [2,3]. By capturing subtle eye movements, eye-tracking facilitates hands-free control, enhances user experience, and has been widely applied in fields such as virtual/augmented reality (VR/AR), clinical research, and assisted driving [4,5]. More importantly, it plays a crucial role in enabling dynamic, naturalistic interaction, making it particularly valuable for assistive robotic systems.

In industrial and rehabilitation fields, integrating eye-tracking with Assistive Robotic Arms (ARAs) has demonstrated significant advantages. In industrial settings, such as manufacturing and assembly lines, eye-tracking allows workers to control robotic systems efficiently using only their gaze, reducing physical strain and improving productivity. In rehabilitation, eye-tracking-based ARA systems empower individuals with motor impairments to interact with their surroundings more naturally, enabling them to perform essential tasks with minimal physical effort [6]. By providing a seamless and adaptive control mechanism, the combination of eye tracking and ARAs enhances both accuracy and ease of use in these domains [7].

However, existing systems predominantly rely on gaze-based target selection, which often results in interaction inefficiencies such as the “Midas Touch” problem—unintended activations caused by prolonged fixation [8]. This issue arises due to the lack of dynamic adaptability in conventional classification methods, which primarily distinguish eye movement behaviors such as fixation, saccades, and smooth pursuit using predefined thresholds [9]. While threshold-based methods are simple and widely used, they struggle with adaptability in diverse and dynamic environments. Probabilistic models like Hidden Markov Models (HMMs) and Bayesian Decision Theory (I-BDT) offer greater flexibility but remain sensitive to prior assumptions, limiting their robustness. Consequently, achieving accurate and efficient real-time eye movement classification remains a key challenge for dynamic human–robot interaction.

To overcome these challenges, we propose a novel approach that combines improved classification techniques with real-time robotic control. Specifically, this paper makes the following contributions:

1. An Advanced Gaussian Mixture Model–Hidden Markov Model (GMM-HMM)-Based Algorithm for Ternary Eye Movement Classification: A novel algorithm is proposed, integrating a sum of squared error (SSE) metric for improved feature extraction and hierarchical training. This algorithm demonstrates higher accuracy compared to current mainstream methods and is well-suited for use with commercial-grade eye trackers, enabling robust and adaptable ternary eye movement classification.
2. Integration of GMM-HMM with a Robotic Arm for Gaze-Guided Interaction: The proposed algorithm is seamlessly integrated with a robotic arm system, enabling gaze trajectories to directly guide robotic motion. This approach eliminates dependence on graphical user interfaces or static target selection, providing a dynamic and intuitive solution to human–computer interaction. Compared to traditional gaze-based target selection combined with path-planning methods, the proposed algorithm demonstrates a significant advantage in real-time performance. Experimental results validate the robotic arm’s motion trajectories, confirming the feasibility of key performance indicators such as trajectory curvature variation, angular deviation, and path jitter in handling complex tasks. This integration bridges the gap between gaze behavior recognition and practical interaction, offering a robust and efficient framework for dynamic scenarios.

## 2. Related Work

### 2.1. Eye Movement Classification

Eye movement classification algorithms utilize features captured by eye-tracking devices to categorize input data. Threshold-based methods are commonly employed to distinguish fixations from saccades. Techniques like Velocity Threshold Identification (I-VT) set a velocity threshold to differentiate stationary fixations from rapid saccades: Points below the threshold are labeled fixations, while those above are marked as saccades [10]. Dispersion Threshold Identification (I-DT) calculates the dispersion of sample points within a moving window based on their coordinates, separating fixations and saccades using a dispersion threshold [11].

Building on binary classification, the Velocity–Velocity Threshold Identification (I-VVT) method improves upon I-VT by employing two velocity thresholds for ternary eye movement classification. Komogortsev developed the I-VDT algorithm, which uses velocity thresholds to detect saccades and adjusts the dispersion window to differentiate fixations and smooth pursuits based on each movement’s characteristics [12]. However, human vision is influenced by various factors, making threshold determination reliant on statistical analysis and limiting adaptability [13].

Probabilistic algorithms offer more versatility. While threshold-based methods are simple and fast, they require clear distinctions between eye movements, limiting their effectiveness. Probabilistic methods like the Hidden Markov Model (HMM) analyze eye movement patterns probabilistically, modeling gaze transitions with a bimodal HMM [14]. Santini introduced the I-BDT algorithm based on Bayesian decision theory for ternary classification, calculating posterior probabilities using prior and likelihood information and employing the velocity-to-time window shift ratio as a feature for online identification [15]. However, I-BDT’s performance depends on accurate prior and likelihood information; inaccuracies can lead to errors. Berndt proposed the I-VDT-HMM fusion algorithm, extending I-VDT by hierarchically training HMM models using velocity and dispersion as features [12]. Although this approach improved temporal modeling, it lacked effective feature-level fusion during training, and its classification accuracy is sensitive to the chosen distribution of the window duration for dispersion—something that is difficult to optimize in real-world applications. In parallel, deep learning-based models have also emerged. Startsev employed a 1D-CNN with BLSTM for fixation, saccade, and pursuit classification, while Goltz explored simplified neural network architectures for similar tasks [16,17]. These models demonstrate strong performance but generally require large labeled datasets and computational resources, limiting their use in lightweight, real-time systems. In contrast, our proposed GMM-HMM method offers an unsupervised, probabilistic solution that avoids the need for large training datasets and provides interpretable results. By emphasizing model efficiency and real-time capability, it is particularly suitable for gaze-based control in assistive robotic applications, where low latency and robustness are essential [18,19].

### 2.2. Eye Tracking and HCI

Eye movement-based control methods for robotic arms have garnered significant attention in recent years, particularly for assisting individuals with severe physical impairments [20]. The primary approaches in this domain can be broadly categorized into three types: telemanipulation, directional gaze, and object-oriented gaze.

Telemanipulation involves the use of digital interfaces, typically displayed on a screen, where users can control the robotic arm by fixating on specific on-screen buttons [21]. These buttons correspond to different actions, such as moving the arm in various directions or executing tasks like grasping objects. This method is advantageous for users who may not have direct visual access to the robotic arm, such as bedridden patients, as it allows for remote control through a graphical user interface (GUI) [22]. However, the reliance on a screen can sometimes limit the user’s intuitive control in a three-dimensional space [23].

Directional gaze control leverages rapid eye movements (saccades) and fixation to direct the robotic arm’s movement [24,25]. By focusing their gaze in a particular direction, users can command the arm to move correspondingly [26]. This method is often combined with additional inputs like blinks or brain–computer interfaces (BCIs) to enhance control accuracy and system responsiveness [27]. Directional gaze methods are particularly useful for tasks that require the robotic arm to navigate through a space, such as pick-and-place operations. However, translating 2D eye movements into 3D arm movements remains a significant challenge [7].

Object-oriented gaze focuses on the user’s gaze fixation on specific objects to trigger the robotic arm’s actions [28]. This approach integrates computer vision technologies to identify the object of interest and automates the robotic arm’s trajectory planning for tasks like grasping or manipulating the object. Object-oriented gaze control offers a more natural interaction mode, as users typically look at the objects they wish to interact with [29]. This method is highly effective in scenarios requiring precise object manipulation in a three-dimensional environment [30].

Despite the advancements, challenges such as the “Midas Touch Problem,” where unintended commands are triggered by gaze, and the integration of gaze-based control in dynamic environments remain [31]. The development of multimodal systems that combine gaze with other inputs, such as head movements and voice, shows promise in overcoming these limitations. The introduction of algorithms like the GMM-HMM aims to further refine the accuracy and reliability of gaze-controlled robotic arms, enabling more intuitive and effective human–robot interactions.

## 3. GMM-HMM for Eye Movement Classification

### 3.1. GMM-HMM Model Framework for Gaze Extraction

Eye movement during human–computer interaction demonstrates implicit associations between gaze behavior and interaction goals. When multiple gaze points cluster in a specific area or exhibit a clear pattern, they often imply a specific intended interaction. Spatiotemporal features and velocity characteristics in eye movement data are instrumental in depicting these behaviors. This study utilizes a GMM-HMM model for robust eye movement modeling [32]. In this context, the classification of eye movements into fixation, saccades, and smooth pursuit can be formulated as a three-state first-order HMM problem.

The Gaussian Mixture Model (GMM) aims to represent an *N*-dimensional dataset as a mixture of finite multivariate Gaussian distributions. The probability density function of an order-GMM is given as follows:

(1) $$P\left(x|\mu ,\Sigma \right)=\sum_{k=1}^{K}c_{k}N\left(x|\mu_{k},\Sigma_{k}\right)$$

where $c_{k}$ is the mixture coefficient, summing to 1, and $N(x|\mu_{k},\Sigma_{k})$ represents a multivariate Gaussian distribution with mean vector $\mu_{k}$ and covariance matrix $\Sigma_{k}$ for component *k*. The initialization of GMM parameters is performed using the K-means algorithm, while the Expectation-Maximization (EM) algorithm optimizes the parameters [33].

The Hidden Markov Model (HMM), meanwhile, is a statistical model based on a hidden-state Markov process [34]. It utilizes three parameters, the initial probability matrix, the state transition probability matrix *A*, and the emission probability matrix *B*, which denote the underlying system probabilities. The emission matrix relates observed data to the latent states, and the parameters are estimated using the Baum–Welch algorithm to maximize the likelihood iteratively. Finally, to classify eye movements, the Viterbi algorithm identifies the most likely sequence of hidden states, efficiently distinguishing between fixation, saccades, and smooth pursuit.

Eye movement data consists of multidimensional sequences incorporating time, coordinates, and velocity, each exhibiting distinct statistical features. Gaussian distributions are commonly used to model these latent eye movement states due to their flexibility in handling noisy data [14,33]. Gaussian Mixture Models, in particular, excel in modeling continuous motion, such as eye trajectories. By integrating GMMs with HMMs, this hybrid model can effectively capture the relationships between observed data and hidden states, outperforming traditional HMM methods in terms of adaptability and accuracy.

**Figure 1:**
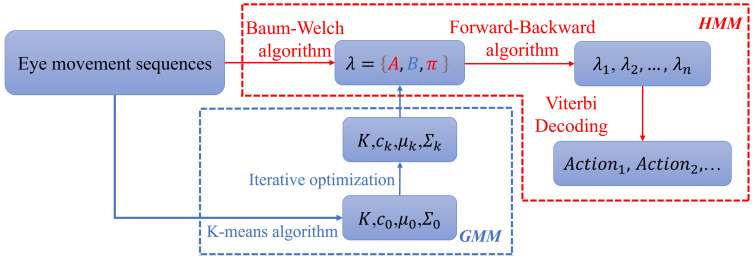
Overview of the proposed GMM-HMM framework for eye movement classification.The diagram illustrates the overall structure integrating Gaussian Mixture Models (GMMs) for feature modeling and Hidden Markov Models (HMMs) for sequential state estimation.

Figure 1 illustrates the GMM-HMM model framework, which serves as a solution to the challenges present in ternary eye movement classification. One notable challenge is the difficulty in distinguishing smooth pursuit behaviors from fixations and saccades, as the former lacks distinct positional and velocity markers. Traditional threshold-based methods such as I-VT and I-DT fall short in handling the variability inherent in human behavior [12]. In contrast, the GMM-HMM framework, enhanced with a sum of squared error (SSE) metric, introduces a path segmentation strategy to address this problem effectively [35].

### 3.2. Eye Movement Path Segmentation

Directly applying the GMM-HMM algorithm to unsegmented eye movement data often yields suboptimal results, partly due to overlapping spatial features that confuse the classification process. To mitigate this, we introduce a novel segmentation approach that enhances feature extraction by focusing on the velocity characteristics of the data.

The proposed segmentation method employs the elbow method to determine optimal segmentation points in the gaze path. Fixation points serve as natural anchors due to their dense spatial distribution. By partitioning the gaze path using these fixation points, segmentation allows for more targeted analysis and clearer differentiation between gaze behaviors, enhancing classification accuracy. Algorithm  1 presents the pseudocode for calculating SSE values using the K-means clustering algorithm [35].

**Figure 2:**
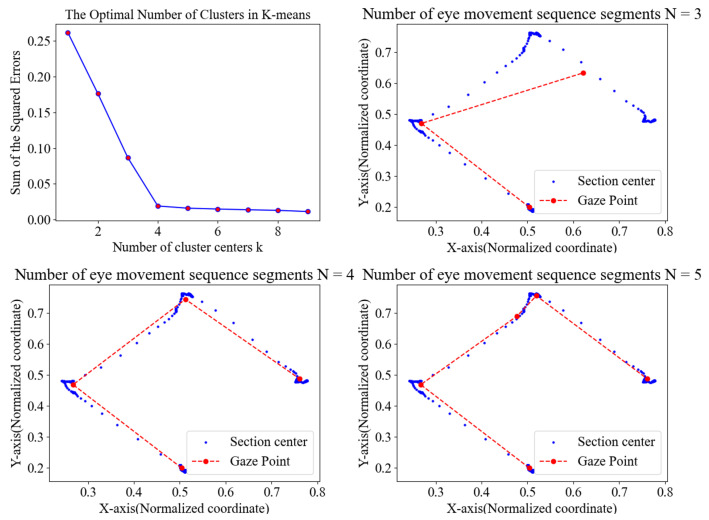
Eye movement sequences segmentation results. The gaze data is partitioned into sub-paths based on the spatial distribution and sum of squared error (SSE) criteria. This segmentation enables more accurate downstream classification by isolating homogeneous eye movement behaviors.

The elbow method is used to determine the ideal number of clusters, where the SSE metric quantifies clustering quality. Higher SSE values signify greater dispersion within clusters, indicating less efficient partitioning. The number of clusters *k* is pivotal in guiding subsequent GMM-HMM classification. As shown in Figure 2, for eye movement data with four fixation behaviors, selecting an appropriate *k* ensures effective segmentation and minimizes ambiguity between behaviors.
**Algorithm 1** Kmeans-SSE**Require:** Eye Movement Sequence Dataset $R^{n}\left(x,y\right)$, Maximum Number of Cluster Centers $K_{m}$, Maximum Iterations max_iter**Ensure:** SSE Values Under Different Number of Cluster Centers 1:**for** k=1 to $K_{m}$ **do** 2:    **for** i=1 to max_iter **do** 3:        Randomly select *k* cluster centers $\mu_{1},\mu_{2},\ldots ,\mu_{k}$ in $R^{n}$ 4:        **for** i=1 to *n* **do** 5:           $c^{\left(i\right)}=$ Index of each point to the nearest $\mu_{i}$ 6:        **end for** 7:        **for** i=1 to *k* **do** 8:           $\mu_{i}=$ Mean value of all points included in cluster center *i* 9:        **end for**10:        Calculate the cost function $J(c^{\left(1\right)},\ldots ,c^{\left(n\right)},\mu_{1},\ldots ,\mu_{k})$11:    **end for**12:    $SSE=\sum_{i=1}^{k}\sum_{p\in C^{i}}{∥p-\mu_{i}∥}^{2}$13:**end for**

### 3.3. Hierarchical GMM-HMM Algorithm Implementation

A direct application of GMM-HMM for eye movement classification may lead to errors, particularly in distinguishing smooth pursuit from other behaviors. To address this, we propose a hierarchical approach involving staged GMM-HMM classification that employs distinct features at each stage.

Initially, the eye movement data undergoes preprocessing using K-means to calculate SSE and determine cluster numbers. This segmentation generates sub-paths, reducing complexity for the first layer of classification. The GMM-HMM model is then applied in two stages: the first layer focuses on the coarse classification of the segmented data using coordinates as features, while the subsequent stage uses velocity information for a finer classification.

**Figure 3:**
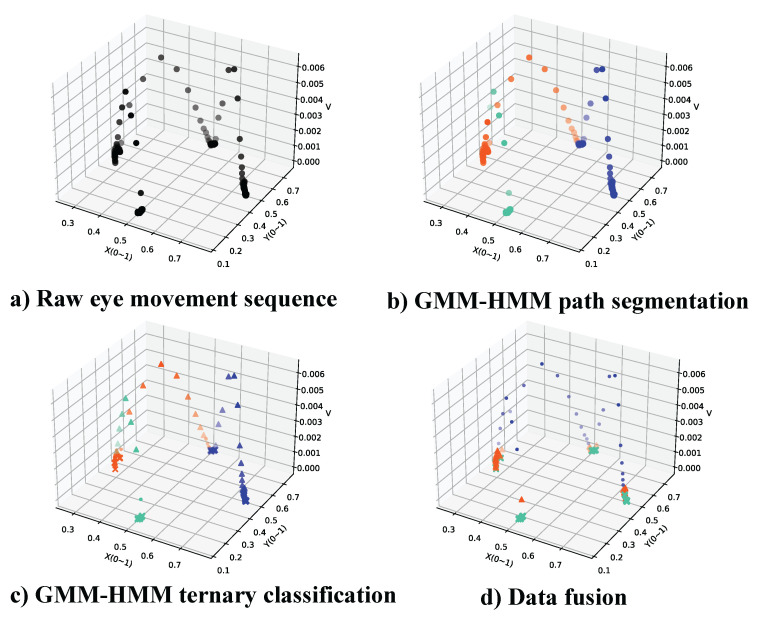
Visual pipeline and results of the hierarchical GMM-HMM classification. (**a**) Raw eye movement data; (**b**) path segmentation using Kmeans-SSE; (**c**) ternary classification into fixations, pursuits, and saccades; (**d**) final fused sequence. The approach combines spatial and velocity features in a two-stage process to improve classification robustness.

**Figure 4:**
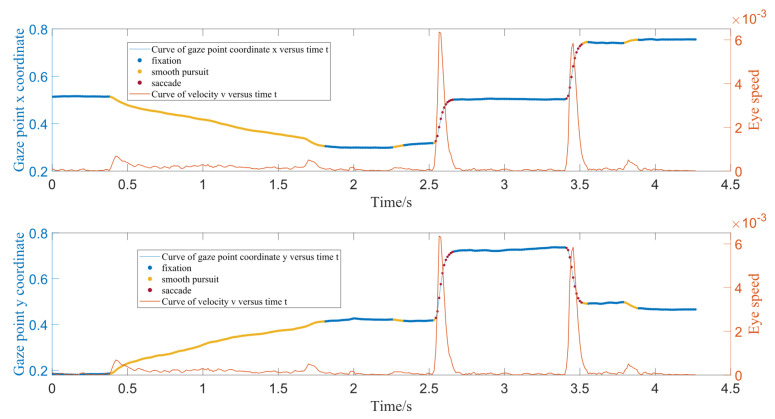
Example of classified eye movement behaviors over a realistic sequence. Colored dots represent different gaze behaviors: blue for fixations, yellow for smooth pursuits, and red for saccades. The model successfully identifies transitions between behaviors and captures subtle gaze drifts, such as the one occurring at 3.6 s.

This hierarchical structure enables a more precise classification process, as spatial features dominate in the initial stage, while velocity features provide refined differentiation in the subsequent stage. Figure 3 illustrates the complete flow of the classification algorithm, including the pseudocode. The key advantages of this approach include its adaptability, as it does not require extensive training datasets, and its resilience, achieved through the incorporation of GMM to model complex distributions. To further demonstrate the effectiveness of this method, we apply it to a real-world example of eye movement data. In Figure 4, a 4.5 s eye movement sequence illustrates the x-coordinate and velocity of gaze points. The sequence includes a 1 s smooth pursuit (yellow dots, 0.5 s to 1.5 s), three 1s fixation periods (blue dots), and two saccades at 2.5 s and 3.5 s (red dots). This example captured a slight gaze drift at 3.6 s, which was classified as smooth pursuit. The classification results, represented by different colors, are superimposed on the original sequence to visually distinguish the three eye movement behaviors.

Algorithm  2 provides a pseudocode example of the hierarchical GMM-HMM algorithm, highlighting the sequence of operations from segmentation to final classification. Compared to single-layer HMMs or threshold-based methods, this hierarchical GMM-HMM strategy offers distinct advantages: 1. It eliminates the need for arbitrary threshold selection by adapting dynamically to the data, ensuring robustness across different subjects and conditions; 2. The use of a multi-layer classification technique effectively captures both spatial and velocity-based features, improving overall performance in gaze behavior classification.
**Algorithm 2** Hierarchical GMM-HMM based on SSE**Require:** Eye Movement Sequence $R^{n}\left(x,y,v\right)$, Original Probability Matrix π, Transition Probability Matrix A, Emission Probability Matrix B, Maximum Iterations $n\_iter_{n}$**Ensure:** Results of Ternary Eye Movement Classification 1:**Step 0: Pre-processing** 2:Execute Kmeans-SSE algorithm to compute the number of optimal path segments *k* 3:**Step 1: First round of GMM-HMM classification** 4:Initialize the parameters (x,y,v) of the first GMM-HMM for selected features 5:**for** i=1 to $n\_iter_{n}$ **do** 6:    Viterbi algorithm 7:    Baum-Welch algorithm 8:    Classify eye movement sequences into *k* clusters 9:**end for**10:**Step 2: Second round of GMM-HMM classification**11:**for** k=1 to *K* **do**12:    Initialize the parameters (x,y,v) of the second GMM-HMM for selected features13:    **for** i=1 to $m\_iter_{2}$ **do**14:        Viterbi algorithm15:        Baum-Welch algorithm16:        Perform ternary eye movement classification17:        Save the classification results of the current sequences states[k]18:    **end for**19:**end for**20:**Step 3: Data fusion**21:Reorganize the classified points into a complete sequence22:**return** List of the classified sequence

## 4. Experimental Setup and Comparative Analysis

To validate the proposed GMM-HMM algorithm, we first conducted a controlled experiment to evaluate its ability to classify eye movement behaviors. This experiment bridges the transition from theoretical model construction to practical validation in human gaze data, establishing a foundation for subsequent robotic application in Section 5.

### 4.1. Data Collection Methods

The experiment was conducted using a computer with a 1920 × 1080 resolution display. A Tobii Eye Tracker 4c (90 Hz sampling rate), manufactured by Tobii, Sweden, was fixed directly below the display to capture eye movements. To minimize the impact of head movements and ensure the accuracy of eye-tracking data, a calibration step was performed before the experiment began. The experimental program included an experimental module for stimuli presentation and a data processing module to handle the collected data [12].

**Figure 5:**
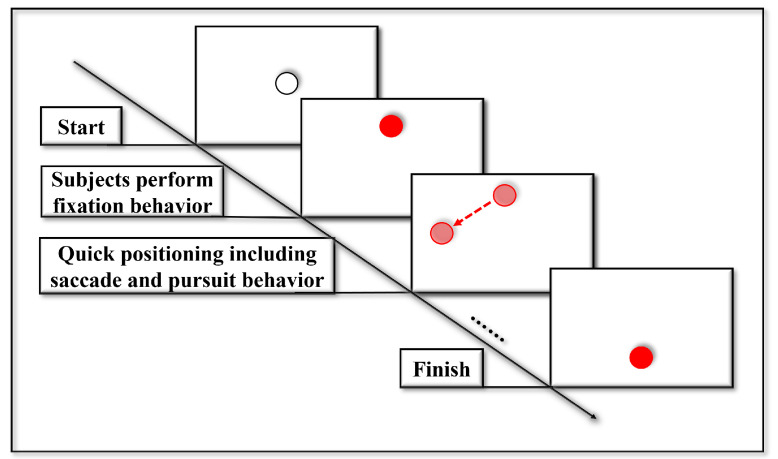
Experimental procedure for data collection using a 2D step target stimulus. A red dot appears in different directions on the screen, prompting participants to shift their gaze. The procedure is designed to elicit fixations, saccades, and smooth pursuits under controlled timing and spatial arrangements.

As shown in Figure 5, the stimulus presented to participants involved a 2D step target, represented by a red dot 80 pixels in diameter. The target appeared in four different directions on the screen, one at a time, for a duration of 1000 ms. Participants were instructed to quickly and accurately gaze at the target as it appeared. The experiment was designed to elicit three types of eye movement behaviors: fixations, saccades, and smooth pursuits. The computer continuously recorded the participants’ gaze data, capturing the location and timing of their eye movements in response to the stimuli.

The dataset for evaluation consisted of eye movement data from 18 participants aged between 20 and 24, all of whom had normal or corrected-to-normal vision. Each participant provided five sets of eye movement data, contributing to a comprehensive dataset. In total, 28,299 fixations, 13,187 smooth pursuits, and 5691 saccades were manually classified based on gaze coordinates and velocity patterns. Fixations were identified by closely clustered points, saccades by large coordinate shifts with high velocity, and smooth pursuits by intermediate velocities with continuous directional trends. The manual annotation process followed the procedure described by Komogortsev, which involved the visual inspection of horizontal and vertical movement components and, in difficult cases, a 3D trajectory view [12]. Although manual labeling may introduce some subjectivity, the annotated dataset has been made publicly available on GitHub to support transparency, reproducibility, and potential further refinement by the research community (https://github.com/lawrence875/eyemovement, accessed on 6 May 2025).

### 4.2. Comparison of Classification Algorithms

This study evaluates the performance of the classification results using four metrics: accuracy, recall, precision, and F1 score. These metrics are defined as follows:

(2) $$\mathrm{Accuracy}=\frac{TP+TN}{TP+FP+TN+FN}$$

(3) $$\mathrm{Recall}=\frac{TP}{TP+FN}$$

(4) $$\mathrm{Precision}=\frac{TP}{TP+FP}$$

(5) $$F1\text{-}\mathrm{score}=\frac{2\times (\mathrm{Precision}\times \mathrm{Recall})}{\mathrm{Precision}+\mathrm{Recall}}$$

where TP, FP, TN, and FN stand for true positive, false positive, true negative, and false negative, respectively. We compare our proposed algorithm with the I-VDT algorithm, chosen for its suitability for low-resolution eye trackers and consistent performance. Additionally, we benchmark against I-BDT, which leverages Bayesian decision theory for robust classification. By comparing with I-VDT and I-BDT, we aim to highlight the strengths and weaknesses of each approach across different methodologies [12,15].

Based on the experimental results of Table 1, the effectiveness of the proposed algorithm for addressing ternary eye movement classification is evident. The obtained accuracy of 94.39% ± 2.08%, precision of 95.31% ± 3.71%, and recall of 94.98% ± 3.93% highlight its ability to accurately classify different eye movement behaviors. When compared to the alternative algorithms, namely I-BDT and I-VDT, our GMM-HMM algorithm showcases superior performance. The reported accuracy, precision, and recall values for I-BDT and I-VDT are notably lower than those achieved by our algorithm. A remarkable aspect of our algorithm is its consistently lower variability, evident in the smaller variances observed in both precision and recall. This indicates that the proposed GMM-HMM algorithm not only achieves higher accuracy but also maintains a more stable performance across multiple evaluations. The reduction in variance underscores the robustness of our approach, making it more reliable for real-world applications. In conclusion, the experimental results substantiate the superiority of our algorithm in effectively addressing the ternary eye movement classification problem.

**Figure 6:**
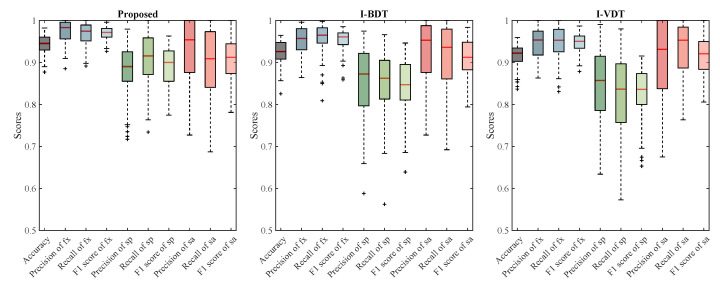
Overall classification accuracy of the proposed algorithm compared with I-VDT and I-BDT. The results are summarized across key metrics, including precision, recall, and F1 score, showing the improved performance of the proposed GMM-HMM method, particularly in smooth pursuit recognition.

We conducted a detailed evaluation of the classification accuracy of our proposed algorithm, I-BDT, and I-VDT for different eye movement behaviors: fixations, smooth pursuits, and saccades. The results highlight the strengths and weaknesses of each algorithm across various metrics, such as precision, recall, and F1 score, as shown in Figure 6 and Table 2, Table 3 and Table 4.

For fixation behavior, our algorithm outperformed both I-BDT and I-VDT in terms of precision, recall, and F1 score. With a precision of 0.9743, our algorithm showed a higher accuracy in correctly identifying fixation events while reducing false positives. In comparison, I-BDT and I-VDT exhibited lower precision scores, suggesting that these algorithms were slightly more prone to misclassifying non-fixation events. The recall of our algorithm was also superior at 0.9665, meaning that it captured more true fixation instances than I-BDT and I-VDT. The balanced F1 score of 0.9699 further highlights the efficiency of our method, showing that it is particularly well-suited for applications that rely on accurate fixation detection. In contrast, while I-BDT and I-VDT demonstrated solid performance, their lower F1 scores (0.9527 and 0.9457, respectively) indicated that they were less effective in balancing precision and recall.

Smooth pursuit has consistently posed challenges in ternary eye movement classification; the proposed algorithm again demonstrated stronger performance with a precision of 0.8784 and recall of 0.9076. These metrics suggest that our algorithm was better at accurately identifying smooth pursuit movements, which involve following moving targets with the eyes. This higher recall indicates that our algorithm effectively minimized false negatives, capturing more true smooth pursuit events compared to I-BDT and I-VDT, which achieved slightly lower precision and recall values. Although I-BDT and I-VDT performed competitively with precision around 0.85 and recall around 0.84, our algorithm’s integration of Gaussian Mixture Models (GMMs) and Hidden Markov Models (HMMs) allowed it to better handle the complexity and continuous nature of smooth pursuit movements, making it more adept at capturing subtle motion transitions.

For saccade behavior, the performance of the algorithms was more balanced. Our algorithm achieved a precision of 0.9301, slightly higher than I-BDT’s 0.9208, indicating the better avoidance of false positives. This suggests that our method is slightly more conservative, which is beneficial in applications where misclassifying non-saccadic movements as saccades could lead to significant errors. However, I-BDT showed a higher recall (0.9135) compared to our algorithm’s 0.8967, meaning that it was better at capturing true saccades, although at the cost of more false positives. I-VDT showed strong performance as well, with a recall of 0.9335 but a slightly lower precision. The resulting F1 scores, which balance precision and recall, were very close, with I-BDT achieving 0.9116 and our algorithm scoring 0.9077, indicating that both algorithms perform similarly well for saccades, each excelling in different aspects of classification.

The nuanced differences in saccade classification between our GMM-HMM and I-BDT stem from the distinct modeling approaches. Our algorithm relies on statistical modeling with hidden states, which is particularly strong at capturing gradual transitions and complex movement patterns, but it may struggle with the rapid, abrupt nature of saccades. In contrast, I-BDT uses Bayesian decision theory, incorporating prior information and likelihood models to handle the quick transitions characteristic of saccades more effectively. This probabilistic approach allows I-BDT to better adapt to fast eye movements but is dependent on the quality of the prior knowledge used.

## 5. Implementation in Robotic Arm Interaction

Following the classification of eye movement behaviors, we integrated the GMM-HMM algorithm into a robotic control system. This section extends the previous evaluation by demonstrating the model’s utility in real-time gaze-guided path planning and manipulation tasks, particularly in assistive scenarios.

### 5.1. System Architecture and Calibration

**Figure 7:**
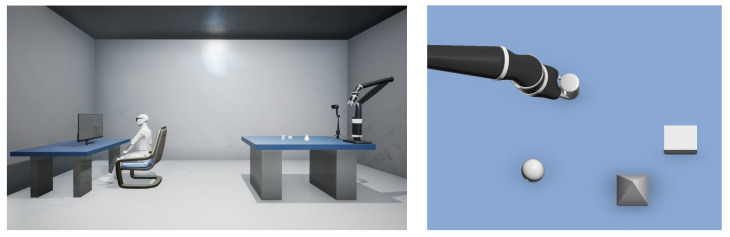
Real-world experimental setup for robotic grasping via gaze. The setup includes a Tobii eye tracker, ZED2 stereo camera, and Kinova robotic arm. The objects on the table are randomly placed to simulate a realistic, unstructured environment for gaze-based robotic control.

The experimental scenario is shown in Figure 7. The experimental setup comprises a depth camera, a desktop eye tracker, and a robotic arm. The depth camera is ZED2, which is both cost-effective and equipped with an array of tools, including camera calibration features, allowing for straightforward user configuration and adjustment. The desktop eye tracker, TOBII’s eye tracker 4c, offers an economical solution with a sampling rate of up to 90 Hz, effectively meeting the experimental demands. The robotic arm, KINOVA GEN2, presents notable cost advantages and is accompanied by an ROS-compatible operation package, facilitating seamless integration with other devices in the experimental framework [36].

This section describes the calibration steps for the system, including camera intrinsic calibration, eye–hand calibration for the robotic arm, and eye tracker calibration, all denoted with $p_{image}$ for consistency. Camera calibration aims to determine the intrinsic parameter matrix *K* using Zhang’s method, which utilizes multiple checkerboard images to estimate *K*. The relation is expressed as follows: $p_{image}=K\left[R|t\right]P_{world}$ For eye–hand calibration, we compute the transformation matrix $T_{camera-hand}$ using Tsai’s method, where $A_{i}T_{camera-hand}=T_{camera-hand}B_{i}$ relates the robotic arm’s motion to the camera’s position [37]. Lastly, the eye tracker calibration maps gaze point coordinates $p_{eye}$ to the pixel coordinates $p_{image}$ via polynomial fitting: $p_{image}=f\left(p_{eye}\right)$ This unified calibration ensures precise spatial mapping and coherence across the system, enhancing experimental accuracy.

**Figure 8:**
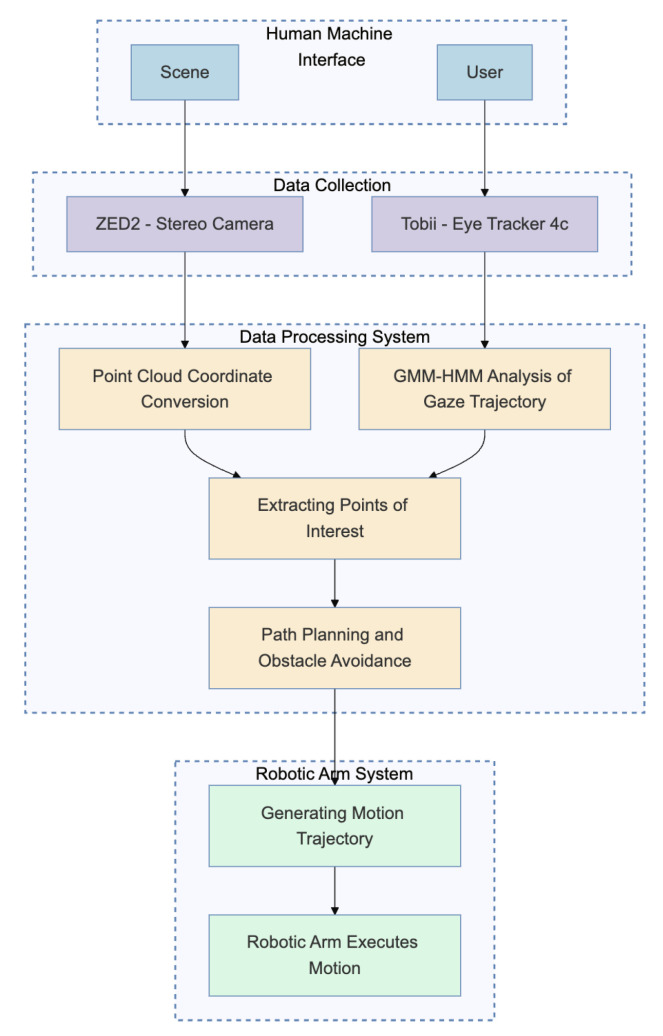
System architecture for gaze-guided robotic grasping. The pipeline integrates gaze data collection, trajectory estimation, coordinate transformation, and real-time execution via ROS. It demonstrates seamless interaction from eye tracking to robotic motion.

Upon completing the system’s calibration, the operational flow of the entire system is illustrated in Figure 8. The system operates on the ROS platform, where the blue frames represent the human–machine interaction interface. Users can observe the working environment through a graphical interface on a host computer, enabling path planning and grasping tasks based on the trajectory of the gaze point. The purple frames indicate the data acquisition devices, which utilize a depth camera to capture RGB images and disparity maps of the working environment. The disparity maps from the stereo cameras facilitate the calculation of the environmental depth, while an eye tracker captures the user’s gaze point coordinates. The yellow frames represent the data processing module, which extracts the user’s gaze point and trajectory through the GMM-HMM algorithm and converts the gaze point coordinates into spatial coordinates within the robotic arm’s coordinate system.

To achieve this conversion, we first define the gaze point in the world coordinate system as follows:

(6) $$P_{\mathrm{world}}={\left[\begin{array}{cccc} X & Y & Z & 1 \end{array}\right]}^{T}$$

Using the camera intrinsic parameters represented by the matrix *K*, along with the extrinsic parameters—specifically the rotation matrix *R* and translation vector *t*—we compute the camera coordinates as follows:

(7) $$P_{\mathrm{camera}}=\left[\begin{array}{cc} R & t\\ 0 & 1 \end{array}\right]P_{\mathrm{world}}$$

The intrinsic parameters of the camera are represented by the matrix *K*, which includes focal lengths $f_{x},f_{y}$ and principal point offsets $c_{x},c_{y}$:

(8) $$K=\left[\begin{array}{ccc} f_{x} & 0 & c_{x}\\ 0 & f_{y} & c_{y}\\ 0 & 0 & 1 \end{array}\right]$$

The conversion of camera coordinates to pixel coordinates is achieved using the following equation:

(9) $$\left[\begin{array}{c} u\\ v\\ 1 \end{array}\right]=K\cdot \left[\begin{array}{c} X_{\mathrm{camera}}\\ Y_{\mathrm{camera}}\\ Z_{\mathrm{camera}} \end{array}\right]$$

Here, ${\left(u,v\right)}^{T}$ denotes the pixel coordinates of the gaze point, and the relationship between the pixel coordinates and gaze point coordinates ${\left(x,y\right)}^{T}$ from the eye tracker is modeled by a mapping function represented by a second-degree polynomial:

(10) $$\left[\begin{array}{c} u\\ v \end{array}\right]=\left[\begin{array}{cccccc} a_{11} & a_{12} & a_{13} & a_{14} & a_{15} & a_{16}\\ a_{21} & a_{22} & a_{23} & a_{24} & a_{25} & a_{26} \end{array}\right]\cdot \left[\begin{array}{c} 1\\ x\\ y\\ x^{2}\\ y^{2}\\ xy \end{array}\right]$$

To reconstruct the 3D point from the pixel coordinates, the pixel coordinates are normalized as follows:

(11) $$x_{\mathrm{norm}}=\frac{u-c_{x}}{f_{x}},\;y_{\mathrm{norm}}=\frac{v-c_{y}}{f_{y}}$$

Subsequently, the ray in the camera coordinate system is expressed as follows:

(12) $$P_{\mathrm{camera}}=Z_{\mathrm{camera}}\cdot \left[\begin{array}{c} x_{\mathrm{norm}}\\ y_{\mathrm{norm}}\\ 1 \end{array}\right]$$

Here, $Z_{\mathrm{camera}}$ (the depth) is obtained from the depth map provided by the stereo camera. Next, the point is transformed from the camera coordinate system to the robotic arm’s coordinate system using the eye–hand calibration transformation matrix $T_{\mathrm{camera}-\mathrm{hand}}$:

(13) $$P_{\mathrm{robotic}}=T_{\mathrm{camera}-\mathrm{hand}}\cdot \left[\begin{array}{c} P_{\mathrm{camera}}\\ 1 \end{array}\right]$$

After converting the user’s gaze point to the target grasping position in the robotic arm’s coordinate system, the arm will sequentially complete the grasping tasks based on the user’s gaze path and the points of interest.

### 5.2. Gaze-Guided Grasping Strategy and Experimental Design

In the experiments, the user controls a robotic arm via eye movements to sequentially grasp objects on a tabletop. The experiment employs two gaze-based interaction methods to guide the robotic arm in path planning and grasping: one approach determines the target position through a stable gaze point, while the other, as proposed in this study, uses the user’s gaze trajectory to guide the path.

In the first approach, the user must maintain focus on the target object’s location on the screen for at least two seconds. Once the system detects this stable gaze point, it designates the location as the target position and initiates path planning algorithms, such as A* or Dijkstra, which segment the scene into a 3D grid using a depth camera to plan the robotic arm’s trajectory [6]. This method captures a stable gaze point to identify the arm’s endpoint for executing the grasping task. In contrast, the approach proposed in this study does not require prolonged focus on a single point. Instead, the system continuously tracks the user’s gaze trajectory and uses it to dynamically guide the robotic arm’s path. When the gaze passes over an object and briefly pauses on it, the GMM-HMM algorithm identifies this pause as the final grasping position. This method enables the user to lead the robotic arm along a desired path through a continuous gaze trajectory, using a short pause on each object to mark specific targets for grasping.

Two experimental setups were conducted: single-object grasping for a target of interest and the sequential grasping of multiple targets. The first setup focuses on comparing the real-time responsiveness and speed of the proposed algorithm against other path planning methods, while the second setup examines the feasibility of using the proposed algorithm to control a robotic arm in complex environments. For experimental consistency, the single-object grasping task was repeated 50 times by a 25-year-old male.

### 5.3. Results and Comparative Analysis

The results presented in the Table 5 highlight the superior performance of the proposed method in single-object grasping tasks. With an average planning time of 2.97 ms, a standard deviation of just 0.83 ms, and a median of 3.0 ms, the method demonstrates remarkable consistency and near-instantaneous response. These attributes are critical for scenarios requiring real-time adjustments, where stability and speed are paramount. In contrast, conventional algorithms such as A* and BiA* exhibit mean planning times of 11.88 ms and 7.55 ms, respectively, with higher variability (standard deviations of 5.17 ms and 3.57 ms). While these times remain acceptable for certain applications, they introduce noticeable delays in high-speed, real-time systems. Dijkstra and Minimum Spanning Tree (MST) both exhibit excessively long planning times, averaging 2829.5 ms and 2869.37 ms, respectively. Their heavy reliance on environmental structure results in significant delays, making them unsuitable for real-time, gaze-controlled grasping tasks. The success rates for all methods are generally high at around 92%, with the proposed approach showing a slightly lower rate of 91%. This minor difference is likely attributable to gaze drift, which may affect the segmentation performed by the GMM-HMM algorithm.

**Figure 9:**
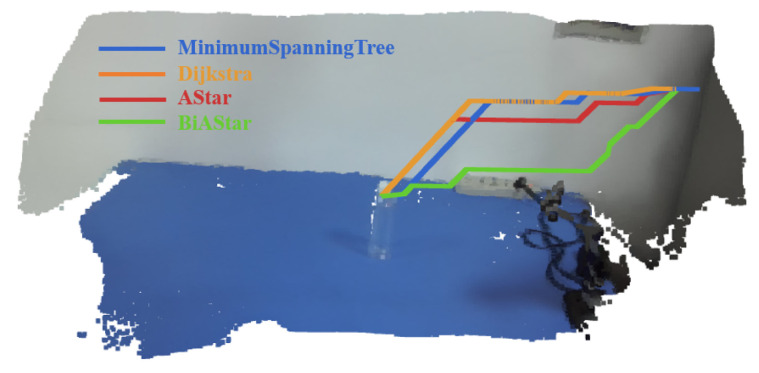
Comparative visualization of path planning performance using traditional methods (A*, BiA*, Dijkstra, and MST) versus the proposed gaze-guided method. The proposed approach shows significantly shorter planning time and improved responsiveness in single-object grasping tasks.

Dijkstra, MST, and A* all rely on the user’s prolonged gaze to determine the target point, with the subsequent path planning handled by traditional 3D spatial algorithms [6,39,40]. This separation makes them highly prone to delays in complex environments, such as maze-like or densely packed point clouds, where their exhaustive search strategies lead to escalating computational costs and inconsistent performance, as illustrated in Figure 9. In contrast, the proposed GMM-HMM method bypasses such limitations by probabilistically modeling the user’s gaze patterns, directly predicting efficient paths irrespective of environmental intricacy. This integration of gaze trajectory and path planning ensures both speed and reliability, making it ideal for real-time gaze-controlled grasping. Unlike traditional methods that rely on separate gaze-based target selection and environment-dependent path planning, GMM-HMM remains robust and efficient, consistently delivering stable performance even in complex scenarios.

In the second set of experiments, multiple objects were arranged for the user to grasp. The user was instructed to sequentially observe the objects with their gaze, while the upper-level control system analyzed the gaze trajectory using the GMM-HMM algorithm to identify points of interest. These points guided the robotic arm to move along the gaze trajectory and execute grasping tasks at the corresponding locations. Additionally, the objects were randomly placed on the experimental table and were not aligned in a straight line. To evaluate the feasibility of the robotic arm’s motion path guided by gaze trajectories, this study calculated key metrics, including the curvature of the motion path and the rate of angular variations in the trajectory.

For a given set of points $p_{i}$, the curvature $\kappa_{i}$ at any point along the motion path is defined as follows:

(14) $$\kappa_{i}=\left\{\begin{array}{cc} \frac{∥v_{1}\times v_{2}∥}{∥v_{1}{∥}^{3}}, & \mathrm{if}\;∥v_{1}∥\;\neq \;0,\\ 0, & \mathrm{else} \end{array}\right.$$

where $v_{1}=p_{i}-p_{i-1},$ and $v_{2}=p_{i+1}-p_{i}$. The rate of angular variation between consecutive tangents at $p_{i}$ can be calculated as follows:

(15) $$\dot{\theta }=\frac{∥v_{1}\times v_{2}∥}{∥v_{1}∥∥v_{2}{∥}^{2}}.$$

**Figure 10:**
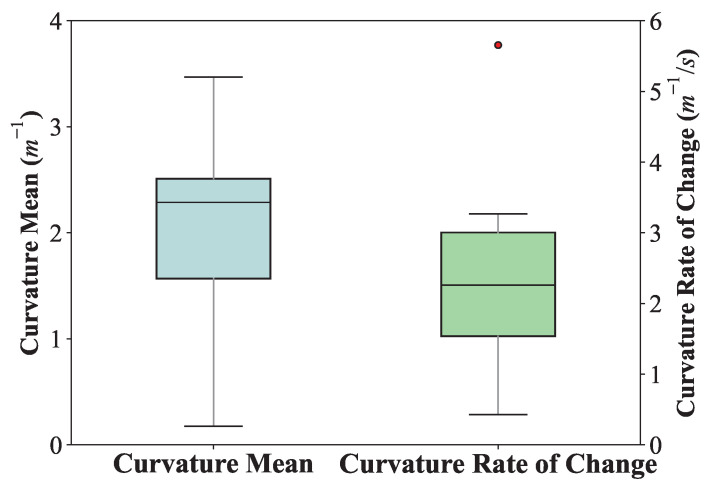
Curvature metrics of robotic trajectories guided by gaze. Boxplots show the distribution of average curvature and curvature rate of change across 20 trajectories. The results indicate smooth, physically feasible motion paths suitable for robotic execution.

Curvature in path planning measures how sharply a trajectory changes direction, directly affecting the smoothness and feasibility of the path [41]. Maintaining appropriate curvature ensures natural, stable motion and avoids impractical or non-physical turns in real-world applications. The referenced study emphasizes that trajectories are smoother and more natural when curvature remains below a threshold of 3 m^−1^ [42]. This aligns with practical requirements in trajectory planning for mobile robots and autonomous systems, where excessive curvature can compromise path feasibility and cause issues in real-world implementations, such as instability or difficulty in path-following [43]. The experiment recorded 20 motion trajectories planned based on gaze trajectories and plotted a boxplot of the mean and standard deviation of curvature changes, as shown in Figure 10.

The results show that the mean curvature values are well below the threshold of 3 m^−1^ [42], indicating that the planned trajectories are smooth and stable. Additionally, the variability in both the mean curvature and the curvature rate of change is moderate, with no extreme deviations except for a single outlier in the rate of curvature change. This consistency further supports the feasibility of the gaze-guided trajectories for real-world applications. The overall smoothness of the paths ensures natural motion, validating the effectiveness of the proposed method.

Robotic motion planning is constrained by maximum allowable angular velocities, which limit rotational speed and ensure mechanical stability during execution [44]. In this study, the mean rate of angular variation in the proposed trajectories was analyzed to assess compatibility with the Kinova JACO2 robotic arm. The average angular variation rate was found to be 0.5864±0.0264rad/s, which is well within the mechanical limits of the JACO2—0.628rad/s for joints 1–3 and 0.838rad/s for joints 4–6. The overall smoothness of the paths ensures natural and stable motion, validating that the generated trajectories can be safely executed on low-cost robotic platforms without exceeding actuator constraints. These results further demonstrate the practicality of the proposed method for real-world robotic applications.

## 6. Limitations and Future Work

Although the proposed algorithm demonstrates robust performance in eye movement classification, the current participant group is limited to young adults aged 20–24. While this ensures visual and motor stability for initial validation, it does not reflect broader population diversity. Future studies will involve participants across different age groups and those with visual or motor impairments to assess generalizability. In addition, the current experiments were conducted in controlled indoor environments with stable lighting and simplified tasks. To further validate the robustness of the system, we plan to evaluate its performance under varying illumination conditions and in more realistic, complex interaction scenarios.

The current system faces several limitations, primarily related to the precision of the camera. When environmental lighting conditions are insufficient, the depth camera struggles to capture the full depth map of the scene, leading to missing depth data. This issue impacts the conversion of the gaze points to 3D points, which, in our experiments, has been identified as a key factor causing failure in robotic arm grasping tasks. Additionally, the eye-tracking trajectory was not filtered during the experiments. Previous studies have shown that applying Kalman filtering to eye movement data can significantly improve the performance of intent recognition tasks [45]. Furthermore, due to gaze drift, Kalman filtering can effectively eliminate erroneous points and enhance the robustness of the entire trajectory.

Future work will focus on two main directions:

1. Improving System Robustness: Efforts will be directed toward enhancing the robustness of the system. This includes applying filtering and compensation techniques to the camera’s point cloud data, as well as employing Kalman filtering and other advanced methods, such as Unscented Kalman Filtering, to filter eye-tracking data. These techniques will help eliminate errors introduced by gaze drift, improving the system’s overall robustness and accuracy.
2. Enhancing Model Capabilities with HMM: The second direction involves leveraging Hidden Markov Models (HMMs) to address evaluation challenges. Specifically, different models will be trained for various eye-tracking trajectories, enabling the system to perform different tasks based on the classified gaze behaviors. While the current approach relies primarily on the decoding capabilities of HMM for classifying eye movements in trajectories, future work will explore combining the two capabilities—trajectory filtering and gaze intent recognition—toward expanding the range of possible applications for this system.

## 7. Conclusions

This study presented a novel approach to enhancing robotic grasp path planning using a Gaussian Mixture Model–Hidden Markov Model (GMM-HMM) algorithm integrated with gaze-based interaction. By addressing the limitations of existing ternary eye movement classification methods, the proposed GMM-HMM framework achieved superior accuracy, precision, and recall, enabling the robust classification of fixation, saccades, and smooth pursuit behaviors. The integration of gaze trajectory-based interaction with a robotic arm system eliminated the need for prolonged fixation or static target selection, offering an intuitive and efficient solution for human–computer interaction. Experimental evaluations demonstrated the system’s ability to achieve dynamic path planning with an average planning time of 2.97 ms, significantly outperforming conventional algorithms such as A* and Dijkstra. Additionally, the proposed system maintained high success rates and smooth motion trajectories across obstacle-free and complex environments, validating its feasibility for real-time applications. Key performance metrics, such as trajectory curvature and angular variation rates, confirmed the stability and adaptability of the planned paths.

This work bridges the gap between gaze behavior recognition and practical robotic control, establishing a robust and time-efficient framework for gaze-guided robotic systems. The proposed solution holds great potential for advancing assistive robotics and dynamic HCI applications, with the adaptability to meet the demands of complex, real-world scenarios.

## Figures and Tables

**Table 1:** Comparison of different classification algorithms.

Algorithm	Accuracy	Precision	Recall	F1 Score
Proposed	μ=94.39%, σ=2.08%	μ=95.31%, σ=3.71%	μ=94.98%, σ=3.93%	μ=94.92%, σ=2.38%
I-BDT	μ=92.43%, σ=2.73%	μ=93.08%, σ=4.52%	μ=93.25%, σ=4.71%	μ=92.92%, σ=3.30%
I-VDT	μ=91.60%, σ=2.77%	μ=92.32%, σ=5.06%	μ=92.42%, σ=4.90%	μ=92.10%, σ=3.30%

**Table 2:** Fixation behavior metrics.

Fixation	Precision	Recall	F1 Score
Proposed	0.9743	0.9665	0.9699
I-BDT	0.9514	0.9553	0.9527
I-VDT	0.9447	0.9487	0.9457

**Table 3:** Smooth pursuit behavior metrics.

Smooth Pursuit	Precision	Recall	F1 Score
Proposed	0.8784	0.9076	0.8893
I-BDT	0.8530	0.8473	0.8445
I-VDT	0.8439	0.8226	0.8271

**Table 4:** Saccade behavior metrics.

Saccade	Precision	Recall	F1 Score
Proposed	0.9301	0.8967	0.9077
I-BDT	0.9208	0.9135	0.9116
I-VDT	0.9094	0.9335	0.9162

**Table 5:** Path planning times for single-object grasping.

Planning Methods	Mean (ms)	Std Dev (ms)	CoV (%)	Median (ms)	Success Rate (%)
Proposed	2.97	0.83	27.81	3.00	91.00
A* [6]	11.88	5.17	43.52	11.15	92.00
BiA* [38]	7.55	3.57	47.28	6.80	90.00
Dijkstra [39]	2829.50	749.38	26.48	2876.90	92.00
MST [40]	2869.37	1044.00	36.39	3465.80	92.00

## Data Availability

All the dataset has been made publicly available on GitHub by URL: (https://github.com/lawrence875/eyemovement, accessed on 6 May 2025).
